# Principles and Technique of Crowns and Bridges

**Published:** 1915-05

**Authors:** 


### BIBLIOGRAPHY.

PRINCIPLES AND TECHNIQUE OF CROWNS AND BRIDGES.—This is a new book on crown and bridge work by Dr. J. F. Hovestadt, Instructor in Crown and Bridge work, Harvard Dental School. The book is well illustrated with well-chosen cuts of the fundamental principles involved, which aid greatly in elucidating the subject without unnecessary redundance in the text. The arrangement of the material is orderly and it is stated with precision that enables one to read it with interest and to follow the details of construction without confusion of mind or technic. The method followed by the author is to clarify the diagnosis by every helpful means available, and to determine the plan of procedure only when the diagnosis has been made clear and complete, a most commendable method of introducing the subject and one too often wholly ignored except as it may be perfunctorily, or so habitually done that it becomes almost automatic, consequently likely to lead to unfavorable results. The diagnosis is followed by a most comprehensive classification of various kinds of crowns and bridges, which makes necessary variations when indicated dependent on fundamental principles of construction which are clearly stated. The subject of oral anesthesia is well presented in its relation to this work, especially as applied to pulp removals, etc. The author details with discretion the various technical procedures of crown making and their combinations into bridges, apparently without emphasizing any particular method of personal preference. The fixed as well as the removable bridges are discussed, giving to each its proper share of commendation. As we view the book, it will be a very useful help to the instructor who will take the trouble to follow it systematically with his class, and amplify those parts which need fuller and at times repeated consideration other than is given in the text. It will be a book of great interest and help to the practitioner who is already more or less familiar with technical details, as he will be unconsciously influenced by the classic treatment of the subject. The book is well printed on excellent paper with good press-work on the cuts. It is published by Ritter & Flebbe, Boston. It may be had of the dental depots and general book stores.

